# Percutaneous dilatational tracheostomy versus surgical tracheostomy in critically ill patients: a systematic review and meta-analysis

**DOI:** 10.1186/cc4887

**Published:** 2006-04-07

**Authors:** Anthony Delaney, Sean M Bagshaw, Marek Nalos

**Affiliations:** 1 Intensive Care Unit, Royal North Shore Hospital, Sydney, NSW, Australia; 2 Department of Intensive Care Medicine, Austin Hospital, Heidelberg, Victoria, Australia; 3 Intensive Care Unit, Nepean Hospital, Penrith, NSW, Australia

## Abstract

**Introduction:**

Tracheostomy is one of the more commonly performed procedures in critically ill patients yet the optimal method of performing tracheostomies in this population remains to be established. The aim of this study was to systematically review and quantitatively synthesize all randomized clinical trials (RCTs), comparing elective percutaneous dilatational tracheostomy (PDT) and surgical tracheostomy (ST) in adult critically ill patients with regards to major short and long term outcomes.

**Methods:**

MEDLINE, EMBASE, CINAHL and the Cochrane Controlled Clinical Trials Register databases were searched to identify relevant studies. Additionally, bibliographies and selected conference proceedings were reviewed, and experts in the field and manufacturers of two PDT kits were contacted. Randomized clinical trials comparing any method of elective PDT to ST that included critically ill adults and reported at least one clinically relevant outcome were included. Data extracted included trial characteristics, measures of study validity, and clinically relevant outcomes.

**Results:**

Seventeen RCTs involving 1,212 patients were included. Most PDTs used a multiple dilator technique and were performed in the intensive care unit (ICU). The pooled odds ratio (OR) for wound infection was 0.28 (95% confidence interval (CI), 0.16 to 0.49, *p *< 0.0005), indicating a significant reduction with PDT compared to ST. Overall, PDT was equivalent to ST for bleeding, major peri-procedural and long-term complications; however, subgroup analysis suggested PDT resulted in a lower incidence of bleeding (OR = 0.29 (95% CI 0.12 to 0.75, *p *= 0.01)) and death (OR = 0.71 (95% CI 0.50 to 1.0, *p *= 0.05)) when the STs were performed in the operating theatre.

**Conclusion:**

PDT reduces the overall incidence of wound infection and may further reduce clinical relevant bleeding and mortality when compared with ST performed in the operating theatre. PDT, performed in the ICU, should be considered the procedure of choice for performing elective tracheostomies in critically ill adult patients.

## Introduction

Tracheostomy, an ancient surgical procedure originally described in the first century BC [1], is one of the more commonly performed procedures in modern intensive care, and is predicted to become more common as demand for intensive care services increases [2,3]. While the benefits of early tracheostomy for patients who require extended periods of mechanical ventilation, compared to prolonged translaryngeal intubation, have been recently debated [4-6], the optimal method of performing tracheostomies in critically ill patients remains unclear.

The traditional method of performing tracheostomies in critically ill patients requires transport from the intensive care unit (ICU) to the operating theatre (OT), where a surgical team performs an open or surgical tracheostomy (ST). This involves a full dissection of the pretracheal tissues and insertion of the tracheostomy tube into the trachea under direct vision [7]. Percutaneous dilatational tracheostomy (PDT) was first described in 1957 [8], and became increasingly popular after the release of a commercially available kit in 1985 [9]. This technique involves the use of blunt dilatation to open the pretracheal tissue for passage of the tracheostomy tube. Proponents of PDT suggest that the limited dissection results in less tissue damage, lowers the risk of bleeding and wound infection, and is able to be performed at the bedside in the ICU, which may overcome the risks associated with transport of critically ill patients to the OT [10].

The proportion of patients receiving PDT and ST varies greatly in different practice settings, with ST being performed exclusively in some ICUs, PDT being performed almost exclusively in others and others using a mixture of both techniques [11-14]. Three previous meta-analyses have attempted to resolve which method of performing tracheostomies is superior; however, results from these reviews are limited for several reasons. One included both randomized and non-randomized studies [15]. The other two meta-analyses included only four [16] and five [17] randomized trials, respectively, made no attempt to find unpublished studies, made no assessment of the possible impact of publication bias, nor explicitly assessed the validity of those trials included. Current guidelines would suggest that meta-analyses lacking these features may arrive at unreliable conclusions [18]. As such, the question of whether PDT or ST is superior for critically ill patients remains unanswered.

To address these shortcomings and provide a comprehensive and contemporary overview, we performed a systematic review and meta-analysis to investigate whether, for adult critically ill patients who require an elective tracheostomy, PDT is superior to ST with regards to the incidence of wound infection, bleeding, and overall mortality as well as major peri-operative complications. We also examined whether one technique was associated with improved rates of longer-term complications.

## Materials and methods

### Search strategy

Randomized clinical trials (RCTs) comparing PDT with ST in critically ill patients were identified using both electronic and manual search strategies supplemented by scanning the bibliographies of all retrieved articles as well as review articles, and reviewing selected conference proceedings (American Thoracic Society (2001 to 2005), Society of Critical Care Medicine (2001 to 2005), European Society of Intensive Care Medicine (2001 to 2005) and American College of Chest Physicians (2002 to 2005)). In addition, we searched for unpublished studies by contacting the manufacturers of two percutaneous tracheostomy kits (Cook^® ^Group Inc, Bloomington, IN, USA) and Smiths Medical (Portex^®^), London, UK), and by contacting experts in the field. All languages were considered eligible. The electronic literature search was completed on December 31, 2005.

MEDLINE (inception to 2005), EMBASE (inception to 2005), CINAHL (inception to 2005) and the Cochrane Controlled Clinical Trials Register databases (inception to 2005) were searched via OVID. MEDLINE (inception to 2005) was also search using the Pubmed interface. Three comprehensive search themes were combined using the Boolean operator 'AND'. The first theme used highly sensitive RCT filters [19,20]. The second theme was created using exploded medical subject headings (MeSH) and textword search for 'tracheostomy' or 'tracheotomy'. The third theme, critical illness, was created by using the Boolean search term 'OR' to search for the following terms appearing as both exploded MeSH and text words: 'critical care' or 'critical illness' or 'intensive care' or 'critically ill'.

### Study selection

An initial screen of all titles and abstracts was conducted to confirm the report was of a trial comparing methods of performing tracheostomies in critically ill patients. The full text articles were retrieved and assessed to determine if they fulfilled the predetermined eligibility criteria for inclusion. Two authors (MN and SB) independently applied the inclusion criteria to the potentially eligible articles, with disagreements resolved by discussion or by resort to a third reviewer (AD). When data were not reported in sufficient detail to determine a studies' eligibility, validity or outcomes, we attempted to contact the corresponding author by email for clarification. The report of one RCT [21] was translated from Korean into English prior to assessment. To be eligible for inclusion the article had to describe a study that fulfilled all of the following criteria: study design – a RCT; intervention – compared any method of elective PDT to ST; population – included critically ill adults; and outcomes – reported at least one of the measures bleeding, wound infection, mortality, duration of mechanical ventilation or ICU length of stay.

### Validity assessment

The validity of the included studies was assessed using *a priori *defined criteria. Each study was assessed for the adequacy of allocation concealment, blinding of outcome assessment, whether the analysis was conducted on an intention-to-treat basis, whether the outcomes were prospectively defined, and whether there were important differences between the two groups at baseline. When details of the allocation concealment were not specified in the article or could not be clarified by contact with the study authors, it was assessed as absent [22]. Blinding was deemed to be present when there was a description for a method of blinded assessment of any of the primary outcomes for that study. Again, two authors (SB and AD) independently assessed the validity of the studies with disagreement resolved by discussion.

### Data abstraction

Data were abstracted onto standardized data collection forms by two authors (SB and AD), independently, with disagreements resolved by discussion. Data were collected regarding patient characteristics, the method of PDT used, the experience of the operators (whether the procedure was performed by a trainee or by an attending/consultant), the location where tracheostomies were performed (ICU or OT), whether the PDTs were performed under bronchoscopic guidance, and the duration of translaryngeal intubation prior to tracheostomy. STs were adjudicated to have been performed in the OT when any of the participants were transferred to the OT to have the procedure performed. Outcome data were collected, including the incidence of bleeding, wound infection, mortality and other major complications of the procedures. When available, data on the duration of mechanical ventilation and duration of ICU stay were recorded. We attempted to include only clinically important outcomes in our analysis. Wound infection was defined variably in the primary RCTs (as shown in Additional file 1), so for our analysis, when possible, only cases that prompted the administration of systemic antibiotics were included as wound infection. Bleeding was defined as bleeding that required an intervention, such as need for blood transfusion or surgical hemostasis, rather than bleeding that resolved spontaneously or with simple pressure. Other major complications were defined as those that were potentially life threatening or required an intervention and included loss of the airway, tube malplacement or pneumothorax. Mortality was defined as all-cause mortality for the longest period of follow-up reported in the study or until hospital discharge. Data were collected on long-term complications when available, including incidence of airway symptoms, delayed closure of fistula, tracheal stenosis, tracheal malacia, and characteristics of the scar.

### Quantitative data synthesis

Agreement on the inclusion of studies was assessed with the Kappa statistic. Statistical heterogeneity was assessed using the χ^2 ^and *I*^2 ^statistics, with an *I*^2 ^value of >50% indicating at least moderate heterogeneity [23]. When no statistical heterogeneity was evident, dichotomous data from selected RCTs were combined using the Mantel and Haenszel method to produce an estimate of the pooled odds ratio (OR) with 95% confidence intervals (CIs). Continuous outcomes were pooled using standardized mean differences (SMDs). The potential for publication bias was assessed by inspection of funnel plots for asymmetry and an Egger's test [24]. *A priori *selected subgroups for sensitivity analysis included two study quality factors (allocation concealment and blinding of outcomes), the method used to perform the PDT, the location where the tracheostomy was performed, and use of bronchoscopic guidance to guide the PDT. All statistical analyses were conducted using STATA 8.2 (StataCorp, College Station, TX, USA).

## Results

### Study selection

**Figure 1:**
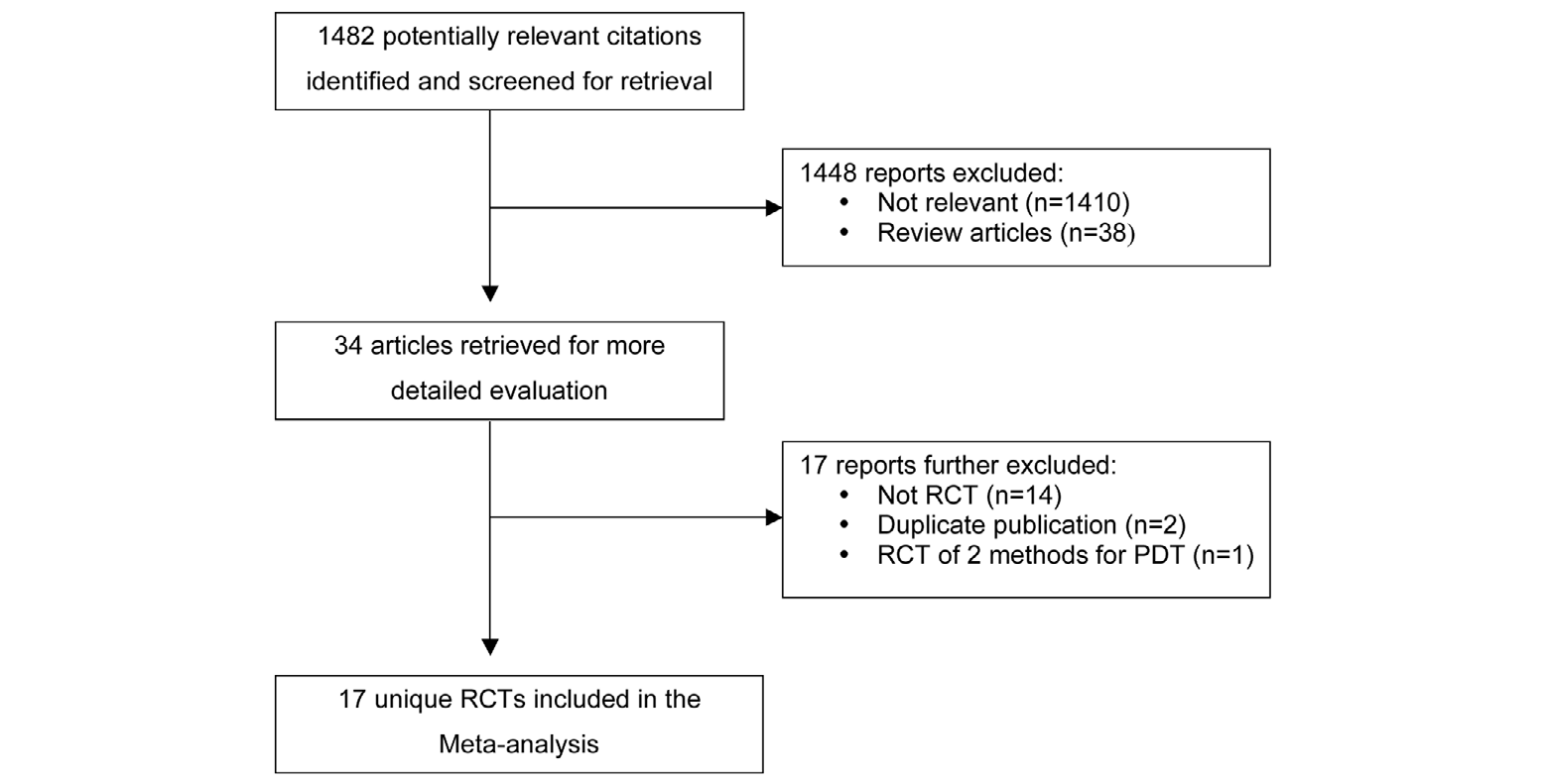
Quorum flow diagram summarizing trial flow and reasons for exclusion of studies. PDT, percutaneous dilatational tracheostomy; RCT, randomized clinical trial.

Database searches generated a total of 1,482 references. There were 34 full text articles retrieved for review with 17 [21,25-38] RCTs fulfilling all eligibility criteria for inclusion in the systematic review. While 16 of the studies were identified by the electronic search strategy, a single study of a recently completed RCT was identified by contact with an expert [39]. In addition, one study was published in abstract form only; however, the authors were contacted and provided complete details of the study, which enabled a thorough review and abstraction of relevant data[35] Agreement on the inclusion studies was 97% (kappa 0.93, *p *< 0.0001). The flow of studies and reasons for exclusion are displayed in Figure 1.

### Study description

A total of 1,212 participants were randomized in the 17 RCTs. The study characteristics are shown in Table 1. The majority (71%) of PDTs used a multiple dilator technique and 94% were performed in the ICU. A summary of the validity assessment for the 17 RCTs is displayed in Table 2. Most studies had balanced groups at baseline and performed their analysis on an intention-to-treat basis; however, only two RCTs used methods to blind the adjudication of outcomes while only seven RCTs clearly maintained allocation concealment. No study had a loss to follow-up of > 5% for the short-term outcomes; however, when longer term outcomes were reported, losses to follow-up were understandably significant, as displayed in Table 3.

### Evidence synthesis

#### Wound infection

**Figure 2:**
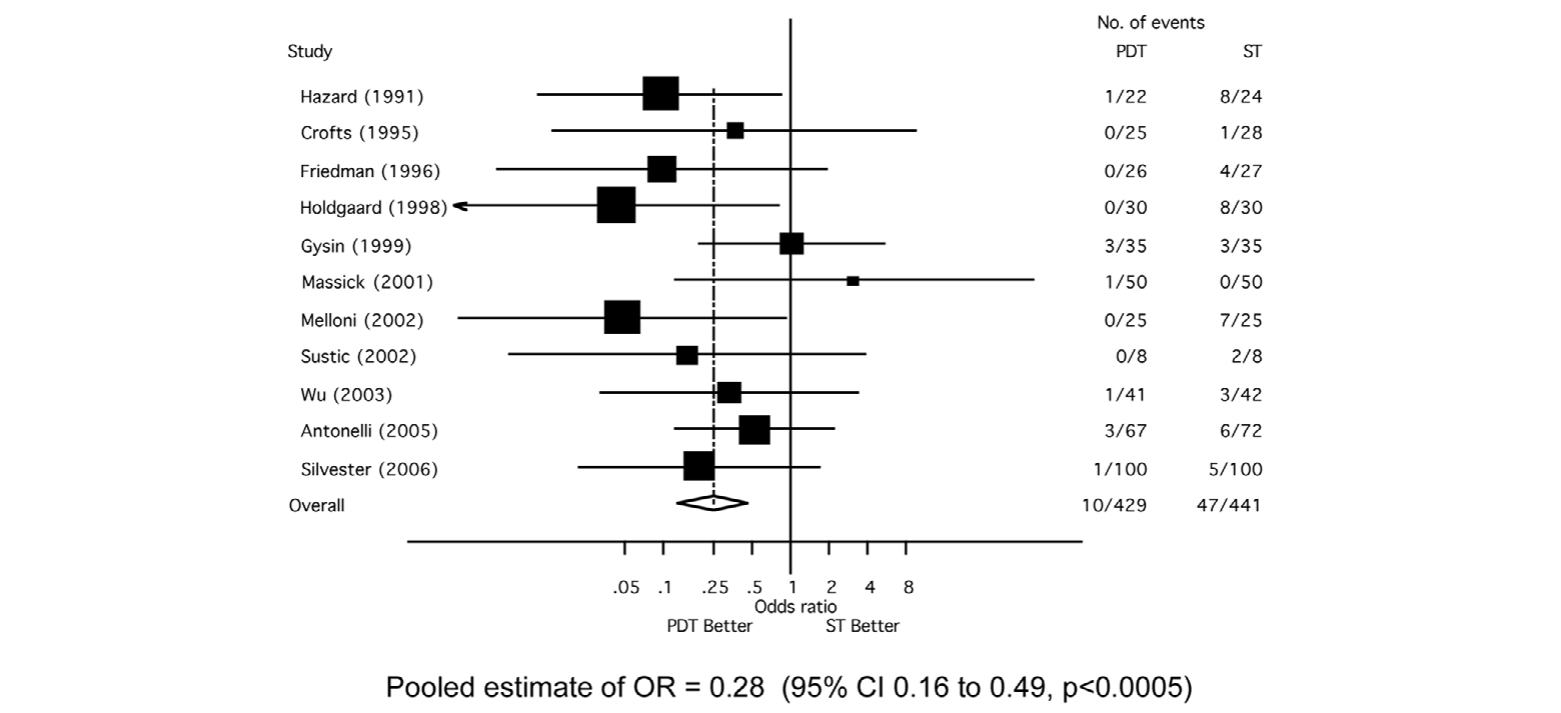
Forest plot showing the effect of percutaneous dilatational tracheostomy (PDT) compared to surgical tracheostomy (ST) on the incidence of wound infection.

Clinically important wound infection was diagnosed in 6.6% (*n *= 57/870) of patients based on data from 11 RCTs [25,26,28-30,32,33,36,38-40] (Figure 2). There was a significant reduction in the OR for wound infection when the tracheostomy was performed using the PDT compared with the ST technique (OR = 0.28; 95% CI, 0.16 to 0.49, *p *< 0.0005). There was no evidence of statistical heterogeneity across studies (χ^2 ^*p *= 0.43, *I*^2 ^= 1.0%) or evidence of bias on inspection of the funnel plot (Additional file 2) or with Eggers test (*p *= 0.18).

#### Bleeding

**Figure 3:**
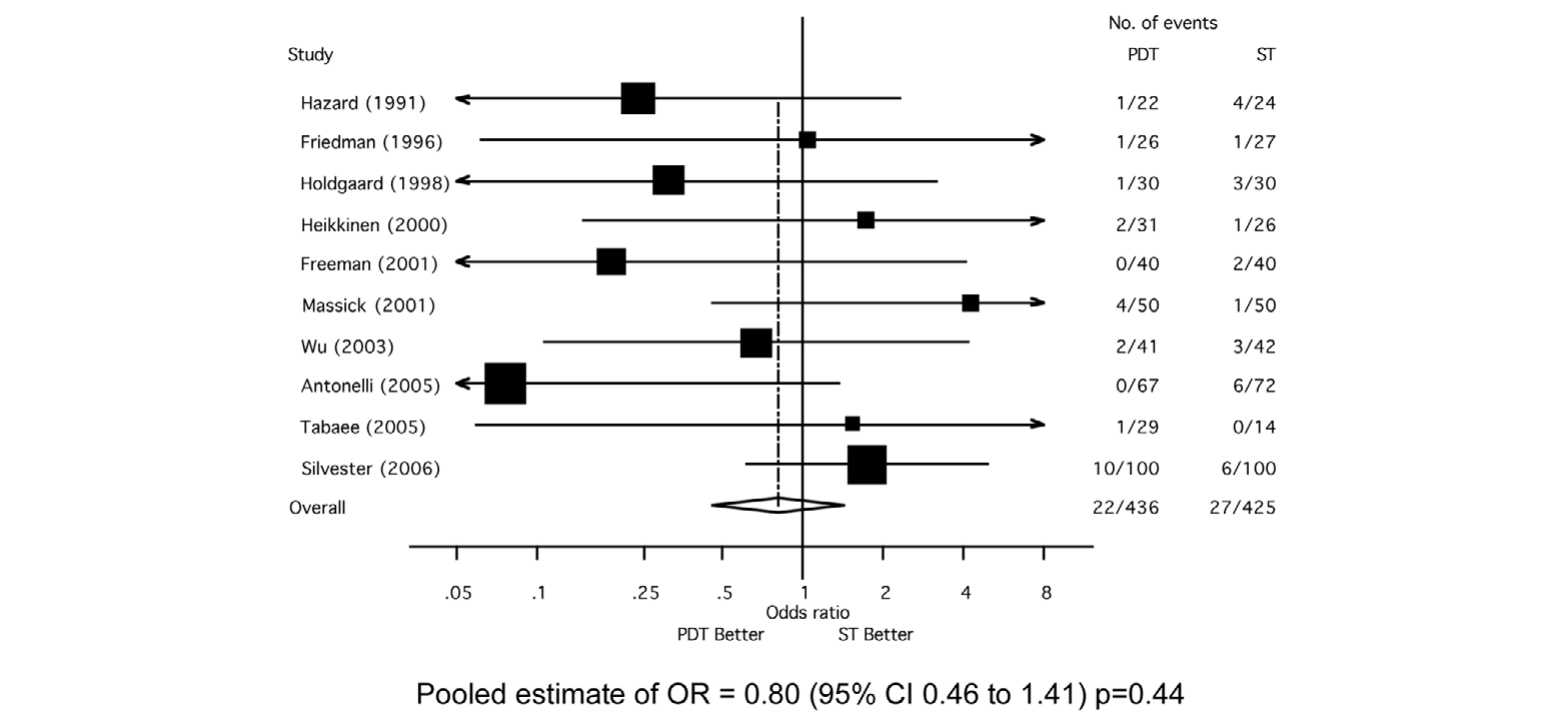
Forest plot showing the effect of percutaneous dilatational tracheostomy (PDT) compared to surgical tracheostomy (ST) on the incidence of significant bleeding.

The overall incidence of clinically relevant bleeding was 5.7% (*n *= 49/861) based on data available from 10 RCTs [25,27,28,30-32,37-40] (Figure 3). There was no significant difference in incidence when comparing PDT to ST (OR = 0.80; 95% CI, 0.45 to 1.41, *p *= 0.35). There was no evidence of significant statistical heterogeneity across studies (χ^2 ^*p *= 0.35, *I*^2 ^= 9.6%) or evidence of bias on inspection of the funnel plot (Additional file 3) or with Egger's test (*p *= 0.14).

#### Mortality

**Figure 4:**
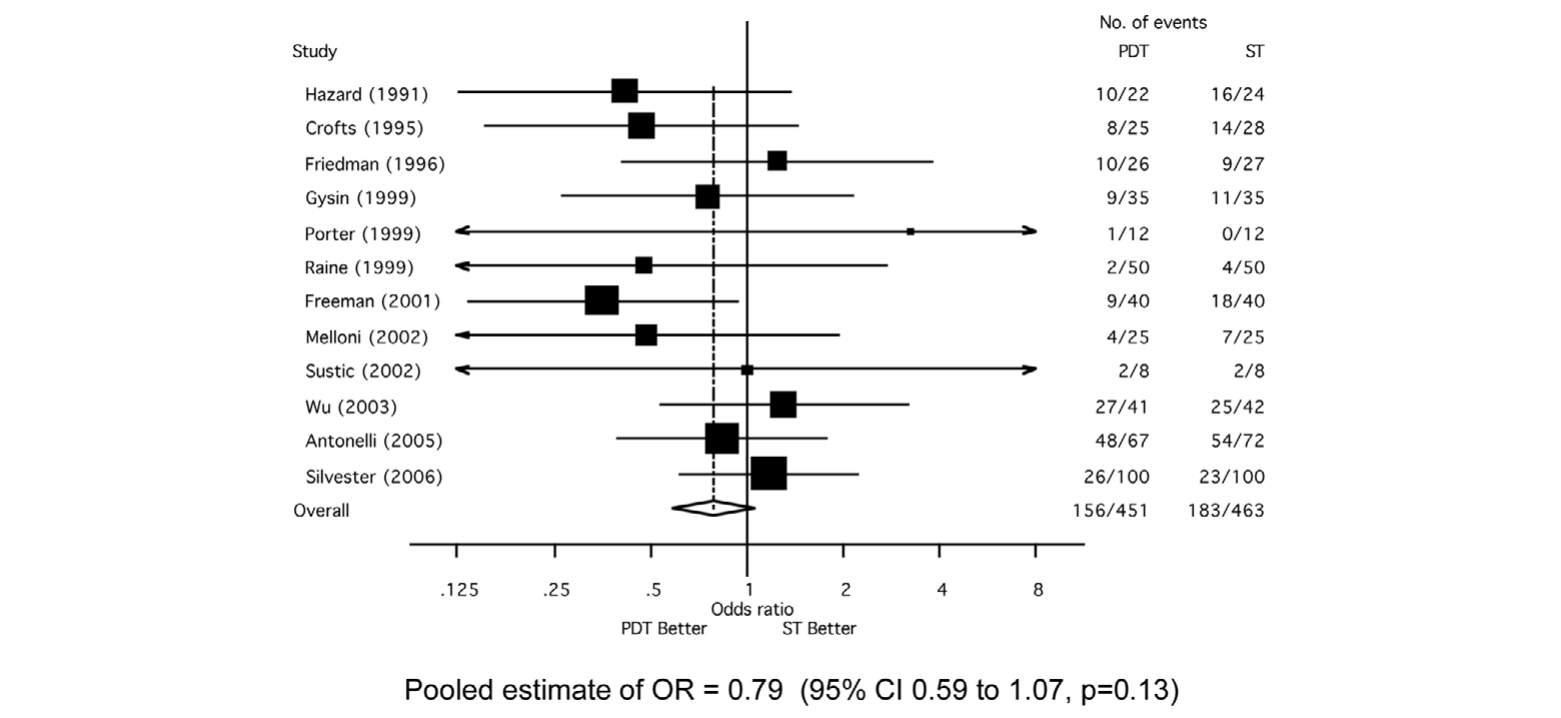
Forest plot showing the effect of percutaneous dilatational tracheostomy (PDT) compared to surgical tracheostomy (ST) on mortality.

The overall mortality rate was 37% (*n *= 339/914) based upon data available from 12 RCTs [25-30,33-35,38,39]. There was no statistically significant difference in mortality for PDT compared with ST (OR = 0.79; 95% CI, 0.59 to 1.07, *p *= 0.13) (Figure 4). There was no evidence of statistical heterogeneity across studies (χ^2 ^*p *= 0.58, *I*^2 ^= 0%) nor evidence of bias by either funnel plot asymmetry (Additional file 4) or Egger's test (*p *= 0.56).

#### Other short-term outcomes

Other major complications occurred in 2.6% (*n *= 15/574) of patients based on data available from 8 RCTs [26,28,30,32,34,37,39,40]. There was no significant difference in the incidence of these major complications between those randomized to PDT or ST (OR = 1.3; 95% CI, 0.50 to 3.42, *p *= 0.59) (Additional file 5). There was no evidence of statistical heterogeneity across studies (χ^2 ^*p *= 0.95, *I*^2 ^= 0%).

The duration of translaryngeal intubation prior to tracheostomy was reported in 15 RCTs [21,25-31,33-39]. There was no difference in the duration of translaryngeal intubation prior to tracheostomy for patients receiving PDT as opposed to ST (Additional file 6). The pooled estimate of the SMD was -0.08 days (95% CI, -0.28 to 0.04, *p *= 0.19). There was no evidence of significant statistical heterogeneity across studies (χ^2 ^*p *= 0.30, *I*^2 ^= 13.4%). The total duration of mechanical ventilation and total ICU length of stay were not sufficiently reported to allow pooling of these results.

### Effect of study quality on major outcomes

Only two RCTs attempted to perform blinding for the adjudication of the presence of wound infection. Therefore, the presence of methods to maintain allocation concealment was the only study quality factor used to assess for influence on the pooled effect estimate. The pooled ORs for wound infection, bleeding and mortality from RCTs where allocation concealment was maintained were not significantly different from the ORs from the RCTs when allocation concealment was not maintained. The results of these analyses are further detailed in Table 4.

### Subgroup analysis

The studies in which the ST was performed in the OT were pooled separately from those where ST was performed in the ICU. There was a significant reduction in the incidence of bleeding with the PDT technique compared to ST, when the STs were performed in the OT (OR = 0.29; 95% CI, 0.12 to 0.75, *p *= 0.01). Interestingly, there was a significant reduction in mortality with the PDT technique, compared to ST, when ST was performed in the OT (OR = 0.71; 95% CI, 0.50 to 1.0, *p *= 0.05). There was a trend toward a shorter duration of translaryngeal intubation prior to tracheostomy with PDT compared with ST, when the ST were performed in the OT (SMD = -0.15; 95% CI, -0.31 to 0.02, *p *= 0.08). Details of the subgroup analyses are shown in Table 4. The method of PDT used and use of the bronchoscope to guide the placement of the PDT did not significantly affect the pooled effect estimates for wound infection, bleeding or mortality.

### Long-term outcomes

Long-term complications for either tracheostomy technique were reported in eight RCTs and are presented in Table 3[25,29-31,33,35,38,39]. Delayed closure of the stoma, airway symptoms, tracheal stenosis and aesthetics of the scar were the most frequently reported complications; however, due to low rates of long-term follow-up, it is difficult to draw definitive inferences from these studies.

## Discussion

This systematic review and meta-analysis has demonstrated that the technique of PDT has a number of important advantages over performing a ST in critically ill patients who require an elective tracheostomy. First, PDT was associated with a reduction in the incidence of clinically important wound infections compared with traditional ST. Secondly, and importantly, there was no evidence overall that PDT resulted in an increased incidence of clinically significant bleeding, major peri-procedural or long term complications. Finally, results of subgroup analysis suggested that PDT was superior to ST when the latter was performed in the OT; specifically, PDT was associated with a reduction in bleeding and overall mortality and a suggestion of decreased duration of translaryngeal intubation prior to tracheostomy.

It is not surprising that a reduced incidence of wound infection was found with the PDT technique. One of the reasons that minimally invasive surgical techniques have become more pervasive in many areas of surgery is the reduction in the rates of surgical site infections [41]. This may be due to minimization of the local tissue damage with a dilatational technique, or may in part be due to a relative preservation of immune functions when minimally invasive techniques are used when compared to an open technique [42].

While there was no statistically significant reduction in mortality with either technique, a possible trend towards lower mortality with use of PDT warrants further discussion. One plausible explanation for this observation could relate to the reduced incidence of infection in those receiving PDT. One study reported the death of a patient who had a ST that could be directly attributable to a local wound infection [28]. The ability to perform the PDT in the ICU without exposing patients to the risks of transport could contribute as well [43,44]. This contention would be supported by the reduction in mortality with PDT when compared to ST performed in the OT. However, this finding is inconclusive and before a change in clinical practice could be recommended, this would need confirmation in a larger, adequately powered multi-center randomized clinical trial.

While subgroup analyses require cautious interpretation in general, the subgroup analysis in this systematic review provides some supportive evidence for the advantages of PDT over ST, in particular when the STs were performed in the OT. One considerable advantage of PDT is the relative safety and convenience of performing the procedure at the bedside in the ICU, which obviates the need to transport a critically ill patient. The transport of critically ill patients is often logistically difficult and exposes the patient to increased likelihood for adverse events and risk to safety [43-46]. The results of our analysis would support the assertion that the elective transport of critically ill patients to the OT for ST may pose undue and increased risk of complications and death.

Additionally, the suggestion of a reduction in the duration of translaryngeal intubation prior to the procedure when comparing PDT with ST performed in the OT may have several important clinical implications. This shorter waiting period prior to performing the PDT may be due to the ease of organizing the PDT at the bedside, specifically forgoing the need for a surgical consultation and the scheduling of time in often busy OTs. Only two studies [28,39] have examined the time taken from the decision to perform a tracheostomy to the procedure being performed. Both found a significantly shorter time when the tracheostomies were performed using the PDT method. This may have additional implications for critically ill patients, including decreased duration of sedation, earlier weaning from mechanical ventilation and shorter overall length of stay in ICU [4,47-49].

While long-term complications appear uncommon, the incomplete follow-up and lack of consistent definitions of outcome measurements in the available RCTs make conclusions difficult to draw with certainty. Non-randomized studies that have examined this issue have found that clinically relevant tracheal stenosis was uncommon in PDT when performed by experienced operators in a long-term study of 326 patients [50]. This experience is not universal [51,52], and further investigation to determine how the method and timing of tracheostomy affects long-term outcomes is warranted.

Several clinical trials have compared the various methods of performing PDT, without any method being shown to be conclusively superior [53-56]. As the majority of studies included in this review used the multiple dilator technique, it is not surprising the results failed to demonstrate any particular benefit from one specific technique of PDT. While it has been suggested that the use of a bronchoscope to guide the operators performing the PDT makes the procedure safer [57], this was not supported by results of our analysis.

There are a number of potential limitations of our review that warrant discussion. Firstly, when considering the above findings, it is important to remember that several groups of patients were excluded from these RCTs, therefore limiting the generalizability of the results of this meta-analysis to all adult critically ill populations. Critically ill patients requiring emergency tracheostomy or with evidence or suspicion of difficult anatomy, prior airway problems, coagulopathies and previous tracheostomy were generally excluded. Thus, ST may still be indicated for selected patients, despite the continuing broader indications for use of PDT [58]. Secondly, while the role of the experience of the operators performing the procedures was presented in Table 1, the effect that the experience of the operators had on the outcomes could not be formally quantitatively assessed in this analysis. Thirdly, there was considerable heterogeneity in the definitions used across studies for the primary outcomes, in particular wound infection and bleeding. We have attempted to compensate for this by only reporting those episodes of wound infection, bleeding or other complications with obvious clinical relevance, requiring an intervention or that resulted in an alteration to patient management. Fourthly, the validity of our conclusions is, in part, dependent upon the validity of the primary RCTs included. Clearly, the practice of blinding is difficult to perform in surgical trials and it may not be possible to completely blind the adjudication of short-term outcomes. One method to partly address this problem is by setting *a priori *definitions for primary outcomes, as was done in some of the RCTs included in this review. In contrast, study design to maintain allocation concealment was conducted or reported in less than half of the reviewed RCTs. Interestingly, however, those RCTs that reported allocation concealment produced results similar to the overall pooled effect estimates. This may suggest that the conclusions of this meta-analysis are relatively robust to the influence of selected study quality factors. Finally, while there was no evidence of potential bias by inspection of the funnel plots, making significant publication bias unlikely, it is possible that studies were not identified for this review that could have had an impact on the pooled effect estimates.

Several important questions remain to be addressed regarding the use of tracheostomies in critically ill patients. While there is divided opinion as to the optimal timing of tracheostomy, there is as yet little definitive evidence to guide clinicians [4]. Similarly, there is a paucity of definitive evidence demonstrating that one technique of PDT is clearly superior to any other. Finally, the feasibility of determining whether any technique of performing a tracheostomy in critically ill patients is superior with regards to long term outcomes must be questionable given the difficulties in obtaining a large enough cohort and adequate follow up to address this issue.

## Conclusion

We have demonstrated that use of PDT is associated with a reduced incidence of wound infection compared to ST in critically ill patients. PDT may yield an overall decreased risk of death when compared with ST. While PDT appears equivalent to ST for the overall incidence of clinically relevant bleeding, major peri-procedural and long term complications, subgroup analysis has revealed that PDT was superior to ST when the ST was performed in the OT. These results indicate that PDT, performed electively in the ICU, should be the method of choice for performing tracheostomies in critically ill adult patients.

## Key messages

• 	PDT is associated with a significantly reduced odds of wound infection compared to ST in critically ill patients.

• 	There was no evidence that PDT was associated with an overall increase in the rate of bleeding, other major complications or long-term complications, compared to ST.

• 	When compared to ST performed in the operating theatres, PDT is associated with a reduced incidence of bleeding, mortality and a trend towards shorter duration of translaryngeal intubation prior to the tracheostomy being performed.

• 	The PDT technique, performed in the ICU, should be considered the technique of choice for critically ill patients who require a tracheostomy.

## Abbreviations

CI = confidence interval; ICU = intensive care unit; OR = odds ratio; OT = operating theatre; PDT = percutaneous dilatational tracheostomy; RCT = randomized clinical trial; SMD = standardized mean difference; ST = surgical tracheostomy.

## Competing interests

The authors declare that they have no competing interests.

## Authors' contributions

AD conceived the study, developed the study protocol, conducted the study search, selected studies, abstracted data, analyzed data, and wrote and revised the manuscript. SMB developed the study protocol, selected studies, abstracted data, analyzed data, and revised and provided critique of successive drafts of the manuscript. MN assisted in developing the study protocol and selected studies and provided critiques of successive drafts of the manuscript. All authors read and approved the final manuscript.

## Supplementary Material

Additional File 1 A table providing a summary of definitions for wound infection for RCTs comparing PDT and ST in critically ill patients. Click here for file

Additional File 2 Funnel plot for the comparison of PDT and ST on the incidence of wound infection. Click here for file

Additional File 3 Funnel plot for the comparison of PDT and ST on the incidence of bleeding. Click here for file

Additional File 4 Funnel plot for the comparison of PDT and ST on mortality. Click here for file

Additional File 5 Forest plot for the comparison of PDT and ST on the incidence of other major complications. Click here for file

Additional File 6 Forest plot showing the comparison of PDT and ST on the duration of translaryngeal intubation prior to tracheostomy. Click here for file

## Figures and Tables

**Table 1:** Characteristics of randomized clinical trials comparing percutaneous dilatational and surgical tracheostomy in critically ill patients

Study	No. of Participants	Mean age (years)	Male (%)	Population	Method of PDT	Location ST performed	Location PDT performed	ST performed by	PDT performed by	Use of bronchoscopic guidance for PDT
Hazard [30]	46	63.1	54.3	Medical/surgical ICU	Multiple dilator	ICU or OT	ICU	Staff	Trainee	No
Crofts [26]	53	59.3	58.5	Medical/surgical ICU	Multiple dilator	OT	ICU	Trainee	Trainee	No
Friedman [28]	53	54.5	58.5	Medical/surgical ICU	Multiple dilator	OT	ICU	Staff	Trainee	No
Holdgaard [32]	60	60	76.7	Medical/surgical ICU	Multiple dilator	OT	OR	Trainee	Staff	No
Jong Joon [21]	38	61	71	Medical ICU	Multiple dilator	ICU	ICU	Trainee	Trainee	Yes
Gysin [29]	70	55.5	NR	Medical/surgical ICU	Multiple dilator	ICU or OT	ICU or OT	Staff	Staff	Yes
Porter [34]	24	44.8	79.2	Surgical ICU	Multiple dilator	ICU	ICU	Trainee	Trainee	Yes
Raine [35]	100	43.1	66	Medical/surgical ICU	Forceps	ICU	ICU	Staff	Staff	No
Heikkinen [31]	57	64.6	70.2	Medical/surgical ICU	Forceps	ICU	ICU	Staff	Staff	No
Freeman [27]	80	63.4	46.3	Medical/surgical ICU	Multiple dilator	OT	ICU	NR	NR	Yes
Massick [40]	100	NR	NR	Medical/surgical ICU	Multiple dilator	ICU	ICU	Trainee	Trainee	Yes
Melloni [33]	50	56.5	62	Medical/surgical ICU	Multiple dilator	ICU or OT	ICU	Staff	Staff	Yes
Sustic [36]	16	36	81.3	Neurosurgical ICU	Forceps	OT	ICU	Staff	Staff	No
Wu [38]	83	68.8	78.3	Medical/surgical ICU	Multiple dilator	OT	ICU	Trainee	Staff	Yes
Antonelli [25]	139	63.5	59.7	Medical/surgical ICU	Translaryngeal	OT	ICU	Staff	Staff	No
Tabaee [37]	43	60.1	39.5	Medical/surgical ICU	Single dilator	ICU	ICU	Trainee	Trainee	Yes
Silvester [39]	200	60.7	68.5	Medical/surgical ICU	Multiple dilator	ICU	ICU	Trainee	Trainee	Yes

ICU, intensive care unit; NR, not reported; OT, operating theatre; PDT, percutaneous dilatational tracheostomy; ST, surgical tracheostomy.

**Table 2:** Summary of validity assessments for RCTs comparing percutaneous dilatational and surgical tracheostomy in critically ill patients

Study	Allocation concealment	Blinding	Intention-to-treat analysis	Predefined outcomes	Baseline Imbalance
Hazard [30]	No	No	Yes	Yes	No
Crofts [26]	No	No	Yes	Yes	No
Friedman [28]	No	No	Yes	Yes	No
Holdgaard [32]	Yes	No	Yes	Yes	No
Jong Joon [21]	No	No	Yes	No	No
Gysin [29]	No	Yes	Yes	Yes	No
Porter [34]	Yes	No	Yes	Yes	No
Raine [35]	Yes	No	Yes	No	Yes
Heikkinen [31]	No	No	Yes	No	No
Freeman [27]	Yes	No	Yes	Yes	No
Massick [40]	Yes	No	Yes	Yes	No
Melloni [33]	No	No	Yes	Yes	No
Sustic [36]	No	No	Yes	Yes	No
Wu [38]	No	No	No	Yes	No
Antonelli [25]	Yes	No	Yes	Yes	No
Tabaee [37]	No	No	Yes	Yes	No
Silvester [39]	Yes	Yes	Yes	Yes	No

ICU, intensive care unit; NR, not reported; OT, operating theatre; PDT, percutaneous dilatational tracheostomy; ST, surgical tracheostomy.

**Table 3:** Summary of long-term complications comparing percutaneous dilatational and surgical tracheostomy in critically ill patients

Study	Proportion available for long-term follow-up (%)		Duration of follow-up	Reported complications		
	ST	PDT		Complication	ST (%)	PDT (%)

Hazard [30]	8/24 (33)	11/22 (50)	1.5–3 months	Delayed closure	3 (38)	0 (0)
				Tracheal stenosis	5 (63)	2 (18)
				Cosmetic deformity	2 (25)	1 (9)
Gysin [29]	20/35 (57)	10/35 (29)	3 months	Delayed closure	2 (10)	1 (10)
				Tracheal cartilage lesion	1 (5)	0 (0)
				Unesthetic scar	8 (40)	2 (20)
Raine [35]	26/50 (52)	24/50 (48)	4 months	Tracheal stenosis	11 (46)	7 (27)
				Scar requiring surgical revision	5 (21)	2 (8)
Heikkinen [31]	11/56 (20)	11/56 (20)	18 months	Delayed closure	1	0
				Airway symptoms^a^	2	2
				Dysphagia	1	0
Wu [38]	12/42 (29)	15/41 (37)	2–4 years	Tracheal malacia	1 (8)	0 (0)
Melloni [33]	13/25 (52)	15/25 (60)	6 months	Tracheal malacia	0 (0)	1 (7)
				Tracheal stenosis	0 (0)	1 (7)
Antonelli [25]	13/72 (18)	18/67 (27)	12 months	Delayed closure	7 (54)	7 (39)
				Airway symptoms^a^	6 (46)	5 (28)
				Tracheal stenosis	2 (11)	1 (6)
				Need for stomaplasty	3 (16)	1 (6)
Silvester [39]	42/100 (42)	29/100 (29)	20 months	Airway symptoms^a^	10 (24)	12 (41)
				Stridor	2 (5)	0 (0)
				Vocal cord paralysis	1 (2)	0 (0)
				Unesthetic scar	2 (5)	0 (0)

^a^Airway symptoms included hoarseness, feeling of a lump in the throat, cough, dyspnea, or subjective phonetic or respiratory problems. PDT, percutaneous dilatational tracheostomy; ST, surgical tracheostomy.

**Table 4:** Sensitivity analysis for primary outcomes comparing percutaneous dilatational and surgical tracheostomy in critically ill patients

Outcome	Subgroup	No. of studies	Estimate of OR	95% CI	Test for between subgroup heterogeneity
					
Infection	Allocation concealment				*P *= 0.65
	Yes	4	0.33	0.14–0.77	
	No	7	0.24	0.11–0.53	
	Method of PDT				*P *= 0.62
	Multiple dilator	9	0.24	0.13–0.47	
	Forceps	1	0.15	0.01–3.74	
	Translaryngeal	1	0.55	0.15–2.1	
	ST performed				*P *= 0.70
	ICU	2	0.49	0.12–1.99	
	OT	9	0.25	0.13–0.47	
	Use of Bronchoscope for PDT				*P *= 0.28
	Yes	5	0.40	0.18–0.89	
	No	6	0.20	0.09–0.46	
					
Bleeding	Allocation concealment				*P *= 0.67
	Yes	5	0.85	0.46–1.41	
	No	5	0.71	0.26–1.94	
	Method of PDT				*P *= 0.33
	Multiple dilator	7	0.96	0.51–1.83	
	Forceps	1	1.7	0.15–20.17	
	Translaryngeal	1	0.08	0.004–1.37	
	Single dilator	1	1.5	0.06–39.24	
	ST performed				*P *= 0.009
	ICU	4	2.01	0.86–4.69	
	OT	6	0.29	0.12–0.75	
	Use of bronchoscope for PDT				*P *= 0.08
	Yes	5	1.36	0.65–2.84	
	No	5	0.34	0.12–0.92	
					
Mortality	Allocation concealment				*P *= 0.86
	Yes	5	0.81	0.54–1.22	
	No	7	0.77	0.50–1.19	
	Method of PDT				*P *= 0.94
	Multiple dilator	9	0.80	0.57–1.11	
	Forceps	2	0.63	0.16–2.45	
	Translaryngeal	1	0.84	0.40–1.79	
	ST performed				*P *= 0.2
	ICU	3	1.09	0.61–1.97	
	OT	9	0.79	0.50–1.00	
	Use of bronchoscope for PDT				*P *= 0.52
	Yes	6	0.86	0.58–1.28	
	No	6	0.71	0.45–1.12	

ICU, intensive care unit; OT, operating theatre; PDT, percutaneous dilatational tracheostomy; ST, surgical tracheostomy.

## References

[B1] Walts PA, Murthy SC, DeCamp MM (2003). Techniques of surgical tracheostomy. Clin Chest Med.

[B2] Cox CE, Carson SS, Holmes GM, Howard A, Carey TS (2004). Increase in tracheostomy for prolonged mechanical ventilation in North Carolina, 1993–2002. Crit Care Med.

[B3] Needham DM, Bronskill SE, Calinawan JR, Sibbald WJ, Pronovost PJ, Laupacis A (2005). Projected incidence of mechanical ventilation in Ontario to 2026: Preparing for the aging baby boomers. Crit Care Med.

[B4] Griffiths J, Barber VS, Morgan L, Young JD (2005). Systematic review and meta-analysis of studies of the timing of tracheostomy in adult patients undergoing artificial ventilation. BMJ.

[B5] Freeman BD, Borecki IB, Coopersmith CM, Buchman TG (2005). Relationship between tracheostomy timing and duration of mechanical ventilation in critically ill patients. Crit Care Med.

[B6] Nieszkowska A, Combes A, Luyt CE, Ksibi H, Trouillet JL, Gibert C, Chastre J (2005). Impact of tracheotomy on sedative administration, sedation level, and comfort of mechanically ventilated intensive care unit patients. Crit Care Med.

[B7] McWhorter AJ (2003). Tracheotomy: timing and techniques. Curr Opin Otolaryngol Head Neck Surg.

[B8] Shelden C, Pudenz R (1957). Percutaneous tracheotomy. J Am Med Assoc.

[B9] Ciaglia P, Firsching R, Syniec C (1985). Elective percutaneous dilatational tracheostomy. A new simple bedside procedure; preliminary report. Chest.

[B10] Al-Ansari MA, Hijazi MH (2005). Clinical review: Percutaneous dilatational tracheostomy. Crit Care.

[B11] Blot F, Melot C (2005). Indications, timing, and techniques of tracheostomy in 152 French ICUs. Chest.

[B12] Fikkers BG, Fransen GA, van der Hoeven JG, Briede IS, van den Hoogen FJ (2003). Tracheostomy for long-term ventilated patients: a postal survey of ICU practice in The Netherlands. Intensive Care Med.

[B13] Fischler L, Erhart S, Kleger GR, Frutiger A (2000). Prevalence of tracheostomy in ICU patients. A nation-wide survey in Switzerland. Intensive Care Med.

[B14] Krishnan K, Elliot SC, Mallick A (2005). The current practice of tracheostomy in the United Kingdom: a postal survey. Anaesthesia.

[B15] Dulguerov P, Gysin C, Perneger TV, Chevrolet JC (1999). Percutaneous or surgical tracheostomy: a meta-analysis. Crit Care Med.

[B16] Cheng E, Fee WE (2000). Dilatational versus standard tracheostomy: a meta-analysis. Ann Otol Rhinol Laryngol.

[B17] Freeman BD, Isabella K, Lin N, Buchman TG (2000). A meta-analysis of prospective trials comparing percutaneous and surgical tracheostomy in critically ill patients. Chest.

[B18] Moher D, Cook DJ, Eastwood S, Olkin I, Rennie D, Stroup DF (1999). Improving the quality of reports of meta-analyses of randomised controlled trials: the QUOROM statement. Quality of Reporting of Meta-analyses. Lancet.

[B19] Haynes RB, Wilczynski N, McKibbon KA, Walker CJ, Sinclair JC (1994). Developing optimal search strategies for detecting clinically sound studies in MEDLINE. J Am Med Inform Assoc.

[B20] Dickersin K, Scherer R, Lefebvre C (1994). Identifying relevant studies for systematic reviews. BMJ.

[B21] Jong Joon A, Koh Y, Jae Yong C, Ki Man L, Park W, Hong SB, Tae Sun S, Sang Do L, Woo Sung K, Kim DS (1998). Comparison of clinical efficacy between percutaneous dilatational tracheostomy and surgical tracheostomy. Tuberculosis Respiratory Dis.

[B22] Schulz KF, Chalmers I, Hayes RJ, Altman DG (1995). Empirical evidence of bias. Dimensions of methodological quality associated with estimates of treatment effects in controlled trials. JAMA.

[B23] Higgins JP, Thompson SG, Deeks JJ, Altman DG (2003). Measuring inconsistency in meta-analyses. BMJ.

[B24] Egger M, Davey Smith G, Schneider M, Minder C (1997). Bias in meta-analysis detected by a simple, graphical test. BMJ.

[B25] Antonelli M, Michetti V, Di Palma A, Conti G, Pennisi MA, Arcangeli A, Montini L, Bocci MG, Bello G, Almadori G (2005). Percutaneous translaryngeal versus surgical tracheostomy: A randomized trial with 1-yr double-blind follow-up. Crit Care Med.

[B26] Crofts SL, Alzeer A, McGuire GP, Wong DT, Charles D (1995). A comparison of percutaneous and operative tracheostomies in intensive care patients. Can J Anaesth.

[B27] Freeman BD, Isabella K, Cobb JP, Boyle WA, Schmieg RE, Kolleff MH, Lin N, Saak T, Thompson EC, Buchman TG (2001). A prospective, randomized study comparing percutaneous with surgical tracheostomy in critically ill patients. Crit Care Med.

[B28] Friedman Y, Fildes J, Mizock B, Samuel J, Patel S, Appavu S, Roberts R (1996). Comparison of percutaneous and surgical tracheostomies. Chest.

[B29] Gysin C, Dulguerov P, Guyot JP, Perneger TV, Abajo B, Chevrolet JC (1999). Percutaneous versus surgical tracheostomy: a double-blind randomized trial. Ann Surg.

[B30] Hazard P, Jones C, Benitone J (1991). Comparative clinical trial of standard operative tracheostomy with percutaneous tracheostomy. Crit Care Med.

[B31] Heikkinen M, Aarnio P, Hannukainen J (2000). Percutaneous dilational tracheostomy or conventional surgical tracheostomy?. Crit Care Med.

[B32] Holdgaard HO, Pedersen J, Jensen RH, Outzen KE, Midtgaard T, Johansen LV, Moller J, Paaske PB (1998). Percutaneous dilatational tracheostomy versus conventional surgical tracheostomy. A clinical randomised study. Acta Anaesthesiologica Scandinavica.

[B33] Melloni G, Muttini S, Gallioli G, Carretta A, Cozzi S, Gemma M, Zannini P (2002). Surgical tracheostomy versus percutaneous dilatational tracheostomy. A prospective-randomized study with long-term follow-up. J Cardiovasc Surg.

[B34] Porter JM, Ivatury RR (1999). Preferred route of tracheostomy--percutaneous versus open at the bedside: a randomized, prospective study in the surgical intensive care unit. Am Surg.

[B35] Raine R, Michell WL, Ruttmann TG, Bloch MB, Thorp M, Gough AM (1999). Late outcome after guide-wire forceps percutaneous tracheostomy – a prospective, randomised comparison with open surgical tracheostomy [abstract]. Br J Anaesth.

[B36] Sustic A, Krstulovic B, Eskinja N, Zelic M, Ledic D, Turina D (2002). Surgical tracheostomy versus percutaneous dilational tracheostomy in patients with anterior cervical spine fixation: preliminary report. Spine.

[B37] Tabaee A, Geng E, Lin J, Kakoullis S, McDonald B, Rodriguez H, Chong D (2005). Impact of neck length on the safety of percutaneous and surgical tracheotomy: a prospective, randomized study. Laryngoscope.

[B38] Wu JJ, Huang MS, Tang GJ, Shih SC, Yang CC, Kao WF, Huang MH, Lee CH (2003). Percutaneous dilatational tracheostomy versus open tracheostomy – a prospective, randomized, controlled trial. J Chin Med Assoc.

[B39] Silvester W, Goldsmith D, Uchino S, Bellomo R, Knight S, Seevanayagam S, Brazzale D, McMahon M, Buckmaster J, Hart G Percutaneous versus surgical tracheostomy: a randomised controlled study with long-term follow-up. Crit Care Med.

[B40] Massick DD, Yao S, Powell DM, Griesen D, Hobgood T, Allen JN, Schuller DE (2001). Bedside tracheostomy in the intensive care unit: a prospective randomized trial comparing open surgical tracheostomy with endoscopically guided percutaneous dilational tracheotomy. Laryngoscope.

[B41] Targarona EM, Balague C, Knook MM, Trias M (2000). Laparoscopic surgery and surgical infection. Br J Surg.

[B42] Iwanaka T, Arkovitz MS, Arya G, Ziegler MM (1997). Evaluation of operative stress and peritoneal macrophage function in minimally invasive operations. J Am Coll Surg.

[B43] Warren J, Fromm RE, Orr RA, Rotello LC, Horst HM (2004). Guidelines for the inter- and intrahospital transport of critically ill patients. Crit Care Med.

[B44] Waydhas C (1999). Intrahospital transport of critically ill patients. Crit Care.

[B45] Beckmann U, Gillies DM, Berenholtz SM, Wu AW, Pronovost P (2004). Incidents relating to the intra-hospital transfer of critically ill patients. An analysis of the reports submitted to the Australian Incident Monitoring Study in Intensive Care. Intensive Care Med.

[B46] Lovell MA, Mudaliar MY, Klineberg PL (2001). Intrahospital transport of critically ill patients: complications and difficulties. Anaesth Intensive Care.

[B47] Arabi Y, Haddad S, Shirawi N, Al Shimemeri A (2004). Early tracheostomy in intensive care trauma patients improves resource utilization: a cohort study and literature review. Crit Care.

[B48] Rumbak MJ, Newton M, Truncale T, Schwartz SW, Adams JW, Hazard PB (2004). A prospective, randomized, study comparing early percutaneous dilational tracheotomy to prolonged translaryngeal intubation (delayed tracheotomy) in critically ill medical patients. Crit Care Med.

[B49] Shirawi N, Arabi Y (2005). Bench-to-bedside review: Early tracheostomy in critically ill trauma patients. Crit Care.

[B50] Walz MK, Peitgen K, Thurauf N, Trost HA, Wolfhard U, Sander A, Ahmadi C, Eigler FW (1998). Percutaneous dilatational tracheostomy – early results and long-term outcome of 326 critically ill patients. Intensive Care Med.

[B51] Dollner R, Verch M, Schweiger P, Deluigi C, Graf B, Wallner F (2002). Laryngotracheoscopic findings in long-term follow-up after Griggs tracheostomy. Chest.

[B52] Norwood S, Vallina VL, Short K, Saigusa M, Fernandez LG, McLarty JW (2000). Incidence of tracheal stenosis and other late complications after percutaneous tracheostomy. Ann Surg.

[B53] Nates NL, Cooper DJ, Myles PS, Scheinkestel CD, Tuxen DV (2000). Percutaneous tracheostomy in critically ill patients: a prospective, randomized comparison of two techniques. Crit Care Med.

[B54] Ambesh SP, Pandey CK, Srivastava S, Agarwal A, Singh DK (2002). Percutaneous tracheostomy with single dilatation technique: a prospective, randomized comparison of Ciaglia blue rhino versus Griggs' guidewire dilating forceps. Anesth Analg.

[B55] Byhahn C, Wilke HJ, Lischke V, Rinne T, Westphal K (2001). Bedside percutaneous tracheostomy: clinical comparison of Griggs and Fantoni techniques. World J Surg.

[B56] Westphal K, Byhahn C, Wilke HJ, Lischke V (1999). Percutaneous tracheostomy: a clinical comparison of dilatational (Ciaglia) and translaryngeal (Fantoni) techniques. Anesth Analg.

[B57] Oberwalder M, Weis H, Nehoda H, Kafka-Ritsch R, Bonatti H, Prommegger R, Aigner F, Profanter C (2004). Videobronchoscopic guidance makes percutaneous dilational tracheostomy safer. Surg Endosc.

[B58] Ben Nun A, Altman E, Best LA (2005). Extended indications for percutaneous tracheostomy. Ann Thorac Surg.

