# Supplementary material for: Percutaneous dilatational tracheostomy versus surgical tracheostomy in critically ill patients: a systematic review and meta-analysis
Source: Crit Care. 2006 Apr 7;10(2):R55. doi: 10.1186/cc4887 (PMC1550905; doi:10.1186/cc4887)
Supplement: Additional File 1 — A table providing a summary of definitions for wound infection for RCTs comparing PDT and ST in critically ill patients. [file cc4887-S1.doc]

**Supplemental File 1:** Summary of Definitions for Wound Infection for RCTs Comparing Percutaneous Dilatational and Surgical Tracheostomy in Critically Ill Patients.

| **Study** | **Definitions for Wound Infection** |
| --- | --- |
| **Hazard [30]** | Presence of cellulitis and/or necrosis of the tissue bordering the stoma |
| **Crofts [26]** | Inflammation and purulent discharge requiring antibiotic therapy |
| **Friedman [27]** | Purulent drainage from the site with ≥ 1cm of surrounding erythema |
| **Holdgaard [32]** | Minor infection: cellulitis in a few millimetres around the stoma without purulent secretion  Major infection: cellulitis in several millimetres around the stoma with purulent secretion |
| **Jong Joon [21]** | Not specified |
| **Gysin [29]** | Stoma infection required antibiotic therapy |
| **Porter [34]** | Cellulitis and purulent drainage from the wound differing in quality from that of sputum |
| **Raine [35]** | Not specified |
| **Heikkinen [31]** | Not specified |
| **Freeman [27]** | Not specified |
| **Massick [40]** | Stoma infection requiring antibiotic therapy |
| **Melloni [33]** | Presence of cellulitis and/or necrosis of the tissue bordering the stoma |
| **Sustic [36]** | Purulent drainage from the stoma |
| **Wu [38]** | Not specified |
| **Antonelli [25]** | Infection: Inflammation and culture-positive purulence  Severe infection: signs of infection and tissue necrosis |
| **Tabaee [37]** | Purulent drainage from the stoma |
| **Silvester [39]** | Minimal: local inflammation  Moderate: local cellulitis or purulence  Severe: necrosis of wound breakdown |
