# Supplementary material for: Percutaneous dilatational tracheostomy versus surgical tracheostomy in critically ill patients: a systematic review and meta-analysis
Source: Crit Care. 2006 Apr 7;10(2):R55. doi: 10.1186/cc4887 (PMC1550905; doi:10.1186/cc4887)
Supplement: Additional File 3 — Funnel plot for the comparison of PDT and ST on the incidence of bleeding. [file cc4887-S3.doc]

**Supplemental File 3:**

Funnel Plot for the Comparison of PDT and ST on the Incidence of Bleeding


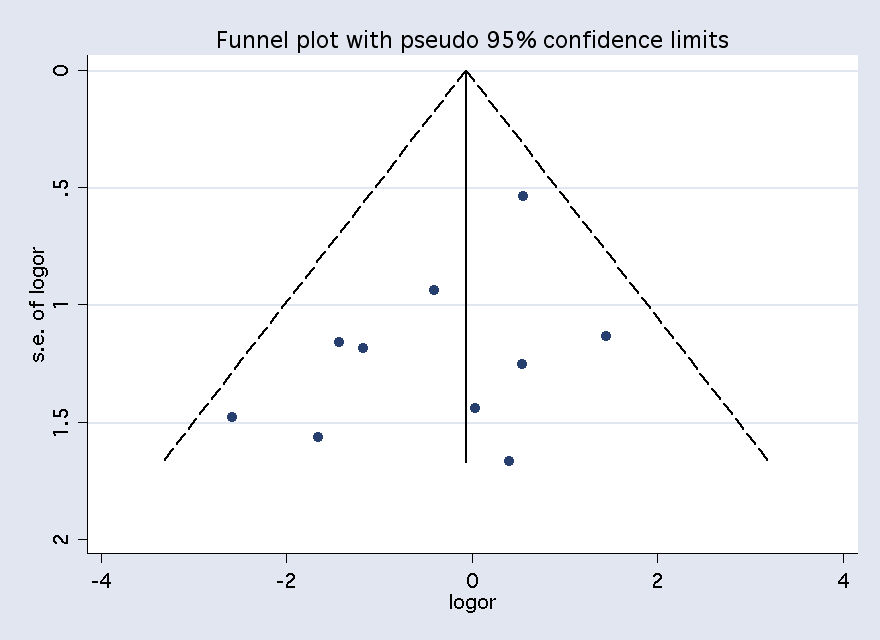


PDT = Percutaneous dilatational tracheostomy

ST = Surgical tracheostomy
