# Supplementary material for: Percutaneous dilatational tracheostomy versus surgical tracheostomy in critically ill patients: a systematic review and meta-analysis
Source: Crit Care. 2006 Apr 7;10(2):R55. doi: 10.1186/cc4887 (PMC1550905; doi:10.1186/cc4887)
Supplement: Additional File 6 — Forest plot showing the comparison of PDT and ST on the duration of translaryngeal intubation prior to tracheostomy. [file cc4887-S6.doc]

**Supplemental File 6.**

Forest Plot Showing the Comparison of PDT and ST on the Duration of Translaryngeal Intubation prior to Tracheostomy

SMD = -0.08 (95% CI, -0.20 to 0.04, p=0.19)

PDT = Percutaneous dilatational tracheostomy

ST = Surgical tracheostomy

ICU = Intensive Care Unit

OR = Operating Room
